# Well prepared: How trichome polymorphism creates an early-warning system against herbivory

**DOI:** 10.1093/plcell/koae253

**Published:** 2024-09-18

**Authors:** Leonard Blaschek

**Affiliations:** Assistant Features Editor, The Plant Cell, American Society of Plant Biologists; Department of Plant & Environmental Sciences, University of Copenhagen, Thorvaldsensvej 40, 1871 Frederiksberg C, Denmark

Herbivores, on their way to take the first bite out of a leaf, will likely encounter trichomes on their path. These small, single- to multicellular organs cover the above-ground surface of many plants and form a physical and phytochemical defense mechanism against herbivores. Recently, Arabidopsis (*A. thaliana*) trichomes were shown to elicit concentric Ca^2+^ waves in response to mechanical deformation, which in turn activated defense-related metabolism (Matsumura et al. 2022). However, while Arabidopsis trichomes are homomorphic, trichomes in many other species, including tomato, appear in strikingly varying shapes and sizes (Wu et al. 2024). The specific roles of these different trichome morphs in herbivore sensing, however, were scarcely understood until now.

In new work, **Chao Sun, JinBo Wei, and colleagues (Sun et al. 2024)** show that the interplay of different trichome morphs creates an early-warning system against herbivory that combines physical barriers with local and systemic metabolic responses. The authors initially observed that caterpillars, when crawling over tomato leaves, would almost exclusively touch long, nonglandular trichomes. That shorter glandular trichomes, despite their established role in defense, did not receive any physical stimulus from the herbivores led the authors to investigate whether there was cross-talk between the different trichome morphs. Indeed, fluorescent calcium imaging showed that caterpillar travel or manual stimulation of long trichomes, even without causing any injury to the plant, triggered Ca^2+^ waves that reached nearby glandular trichomes. Within an hour of stimulating the long trichomes, nearby glandular trichomes upregulated the expression of terpene synthases and jasmonic acid biosynthesis genes, both hallmarks of induced herbivory defense. Not all long trichomes could elicit such responses: it required the presence of basal cells, a specialized cell type at the interface between the trichome and leaf epidermis. Neither long wild-type trichomes without basal cells nor trichomes in a mutant with disrupted basal cell formation could elicit Ca^2+^ waves. In Arabidopsis, trichomes have so-called skirt cells at their base, which were similarly required for Ca^2+^ waves. This observation suggested a conserved role of basal-like cells for trichome calcium signaling.

To pin down the physiological implications of calcium signaling between trichome morphs, Sun et al. (2024) exposed the plants to the cotton bollworm and quantified the effect of mechanical stimulation of trichomes on leaf damage and herbivore development. Wild-type plants that were “primed” for attack by previous mechanical stimulation exhibited less leaf damage and herbivore growth than unprimed plants. In mutants with an increased proportion of long trichomes, both the basal resistance to herbivory and the beneficial effect of priming by mechanical stimulus were greater than in the wild type. Mutationally abolishing long trichomes, or the basal cells of long trichomes, on the other hand, decreased herbivory resistance and negated any positive effects from mechanical priming. Lastly, mutants with only glandular trichomes similarly exhibited severe feeding damage, with no benefit from previous mechanical stimulus. Despite their susceptibility, herbivores feeding on these mutants showed severely reduced growth, even more so when the plants were primed by mechanical stimulation.

**Figure 1.**
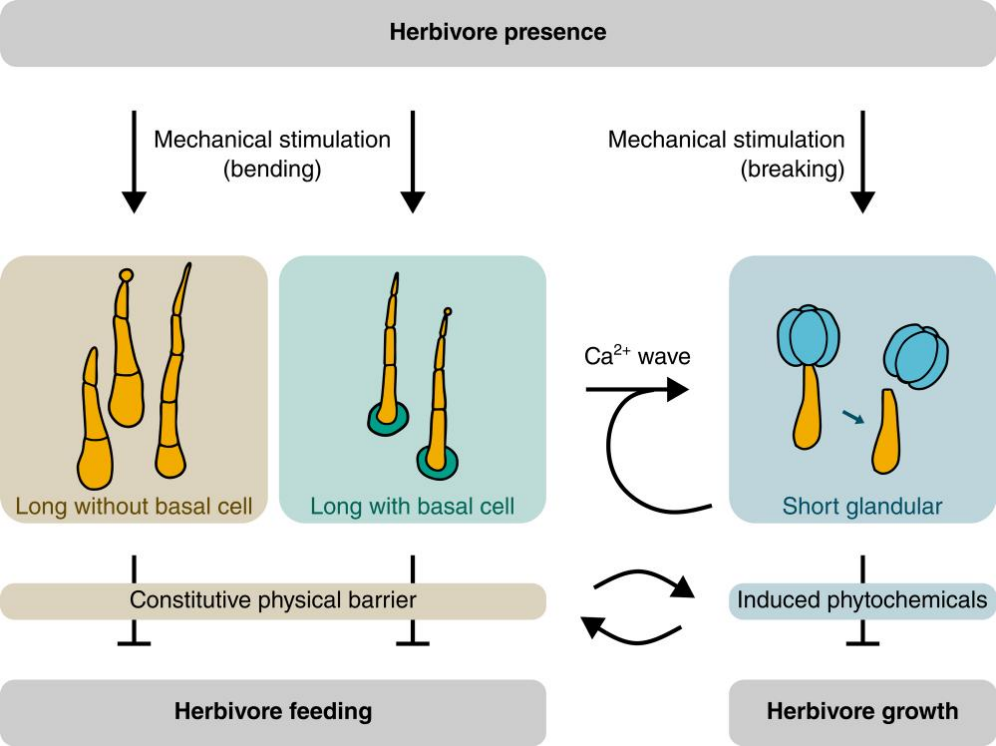
A proposed model of morph-dependent trichome functions in the herbivory defense of tomato. Illustrations adapted from Sun et al. (2024).

Altogether, the results presented by Sun et al. (2024) suggest an approximate model of morph-dependent trichome functions in tomato (Fig. 1): long trichomes function as a constitutive physical barrier to herbivore feeding, and those with basal cells additionally elicit concentric Ca^2+^ waves upon mechanical stimulation. These waves are sensed by nearby glandular trichomes, inducing the accumulation of defense-related phytochemicals such as terpenes. Meanwhile, glandular trichomes that are broken by mechanical stimulation also elicit Ca^2+^ waves. The accumulation of phytochemicals by itself does not deter herbivore feeding, but it significantly impairs herbivore growth. Crucially, considering the severe herbivory damage in mutants with only physical or only chemical defenses, effective herbivore resistance seems to be dependent on synergistic effects between trichome morphs.

Both trichome-triggered Ca^2+^ waves and mechanical stimuli sensed by cells other than trichomes induce additional defenses beyond the metabolism of glandular trichomes (Chehab et al. 2012; Matsumura et al. 2022). However, the precise interplay between constitutive physical barriers and priming of metabolic defenses in glandular trichomes and other leaf cells remains to be fully disentangled. Additionally, the physical barrier function of long trichomes is likely to depend on the specific herbivore tested. Our understanding of the observed synergistic effects between constitutive physical and induced chemical defenses will therefore likely benefit greatly from further studies using additional host–herbivore combinations. Nonetheless, Sun et al. (2024) provide exciting insights into herbivory sensing in tomato, and, from a broader perspective, a great example of functional specificity stemming from minute cellular differences in organ morphs.
